# *N*-(5-Amino-1*H*-tetra­zol-1-yl)formamide

**DOI:** 10.1107/S1600536809044833

**Published:** 2009-10-28

**Authors:** Chun-Lin He, Zhi-Ming Du, Zheng-Qiang Tang, Xiao-Min Cong, Ling-Qiao Meng

**Affiliations:** a State Key Laboratory of Explosion Science and Technology, Beijing Institute of Technology, Beijing 100081, People’s Republic of China

## Abstract

In the title compound, C_2_H_4_N_6_O, the planar [maximum deviation = 0.006 (2) Å] amino­tetra­zole group makes a dihedral angle of 83.65 (8)° with the formamide unit. In the crystal structure, inter­molecular N—H⋯N, N—H⋯O and C—H⋯N hydrogen bonds are responsible for the formation of a three-dimensional network.

## Related literature

For energetic nitro­gen-rich derivatives of 1,5-diamino­tetra­zole, see: Joo *et al.* (2008 ▶). For nitro­gen-rich metastable green chemistry compounds, see: Steinhauser *et al.* (2008 ▶). For 1,5-diamino-1*H*-tetra­zole derivatives, see: Galvez-Ruiz *et al.* (2005 ▶). For the structure of *N*-(1-diacetyl­amino-1*H*-tetra­zol-5-yl)-acetamide, see: He *et al.* (2009 ▶).
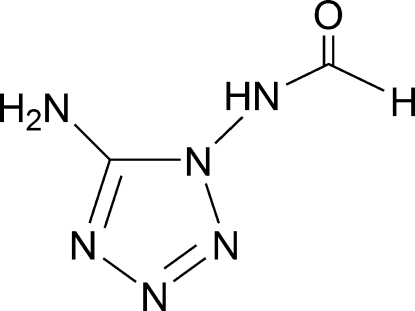


## Experimental

### 

#### Crystal data

C_2_H_4_N_6_O
*M* *_r_* = 128.11
Orthorhombic, 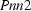

*a* = 10.232 (10) Å
*b* = 12.054 (12) Å
*c* = 4.208 (4) Å
*V* = 519.1 (9) Å^3^
*Z* = 4
Mo *K*α radiation
μ = 0.14 mm^−1^
*T* = 93 K
0.47 × 0.27 × 0.07 mm

#### Data collection

Rigaku Saturn724+ diffractometer
Absorption correction: multi-scan (*ABSCOR*; Higashi, 1995 ▶) *T*_min_ = 0.940, *T*_max_ = 0.991
3464 measured reflections
659 independent reflections
545 reflections with *I* > 2σ(*I*)
*R*_int_ = 0.051

#### Refinement

*R*[*F* ^2^ > 2σ(*F* ^2^)] = 0.036
*wR*(*F* ^2^) = 0.089
*S* = 1.00
659 reflections
94 parameters
1 restraint
H atoms treated by a mixture of independent and constrained refinement
Δρ_max_ = 0.22 e Å^−3^
Δρ_min_ = −0.19 e Å^−3^

### 

Data collection: *CrystalClear* (Rigaku, 2008 ▶); cell refinement: *CrystalClear*; data reduction: *CrystalClear*; program(s) used to solve structure: *SHELXS97* (Sheldrick, 2008 ▶); program(s) used to refine structure: *SHELXL97* (Sheldrick, 2008 ▶); molecular graphics: *ORTEP-3* (Farrugia, 1997 ▶); software used to prepare material for publication: *WinGX* (Farrugia, 1999 ▶).

## Supplementary Material

Crystal structure: contains datablocks I, global. DOI: 10.1107/S1600536809044833/si2213sup1.cif

Structure factors: contains datablocks I. DOI: 10.1107/S1600536809044833/si2213Isup2.hkl

Additional supplementary materials:  crystallographic information; 3D view; checkCIF report

## Figures and Tables

**Table 1:** Hydrogen-bond geometry (Å, °)

*D*—H⋯*A*	*D*—H	H⋯*A*	*D*⋯*A*	*D*—H⋯*A*
N5—H5*N*⋯N1^i^	0.84 (3)	2.02 (3)	2.851 (4)	168 (3)
N6—H6*A*⋯O1^ii^	0.87 (3)	2.54 (3)	2.981 (4)	113 (2)
N6—H6*A*⋯N3^iii^	0.87 (3)	2.35 (3)	3.164 (4)	156 (3)
N6—H6*B*⋯O1^iv^	0.91 (3)	2.12 (3)	3.006 (4)	167 (2)
C2—H2⋯N2^v^	0.95	2.53	3.404 (5)	152

Symmetry codes: (i) 
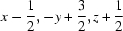
; (ii) 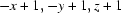; (iii) 
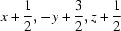
; (iv) 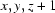; (v) 
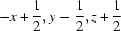
.

**Fig. 1.**
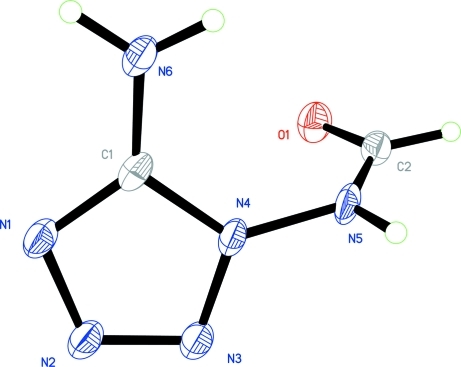
The molecular structure of the title compound with the atom-numbering scheme. Displacement ellipsoids are drawn at the 50% probability level.

## supplementary crystallographic information

### Comment

Nitrogen-containing compounds have received an increasing interest during the last years, these compounds exhibit potential application in gas generator, "green" pyrotechnics and high density energetic materials (Galvez-Ruiz *et al.*, 2005; Steinhauser & Klapötke, 2008; Joo *et al.*, 2008). Rencently, we synthesized a new nitrogen-containing compound, *N*-(5-amino-1*H*-tetrazol-1-yl)formamide, which has a nitrogen content of 65.62%. Herein, we report the crystal structure of the title compound.

The molecular structure of the title compound is presented in Fig. 1. The aminotetrazole group is essentially planar and makes a dihedral angle of 83.65 (8)° with the formamide unit. The bond distances and bond angels in the title compound are similar to the corresponding distances and angles reported by He *et al.* (2009). In the crystal structure, the molecules are linked to each other *via* intermolecular N—H···N, N—H···O and C—H···N hydrogen bonds (Table 1), forming a three-dimensional network structure.

### Experimental

Diamino-tetrazole (10 mmol) was dissolved in 10 ml formic acid, 0.3 g sodium formate was added to the above mixture and reacted under refluxing, use TLC to control the reaction process. After cooling, the crude product precipitated and was filtered. The purity of the compound was checked by its melting point. ^1^H-NMR (DMSO—d_6_, 400 MHz): 11.71(1*H*,s), 8.34(1*H*,s), 7.02(2*H*, s); MS (EI,70 eV) m/*z*:128(*M*^+^). 70 mg of the obtained product was dissolved in the mixture solution of methanol (20 ml) and acetone (10 ml) and the solution was kept at room temperature to give suitable crystals for X-ray structure determination.

### Refinement

Amino H atoms were located in a difference Fourier maps and were refined isotropically. Other H-atoms were placed in calculated positions with C—H = 0.98 Å, and refined in riding mode with *U*_iso_(H) = 1.2*U*_eq_ (C).

In the absence of significant anomalous dispersion effects, the Friedel pairs were averaged.

### Crystal data

**Table:**

C_2_H_4_N_6_O	*F*(000) = 264
*M**_r_* = 128.11	*D*_x_ = 1.639 Mg m^−^^3^
Orthorhombic, *P**n**n*2	Mo *K*α radiation, λ = 0.71073 Å
Hall symbol: P 2 -2n	Cell parameters from 1548 reflections
*a* = 10.232 (10) Å	θ = 3.4–27.2°
*b* = 12.054 (12) Å	µ = 0.14 mm^−^^1^
*c* = 4.208 (4) Å	*T* = 93 K
*V* = 519.1 (9) Å^3^	Platelet, colorless
*Z* = 4	0.47 × 0.27 × 0.07 mm

### Data collection

**Table:**

Rigaku Saturn724+ diffractometer	659 independent reflections
Radiation source: Rotating Anode	545 reflections with *I* > 2σ(*I*)
graphite	*R*_int_ = 0.051
Detector resolution: 28.5714 pixels mm^-1^	θ_max_ = 27.3°, θ_min_ = 3.4°
multi–scan	*h* = −13→12
Absorption correction: multi-scan (*ABSCOR*; Higashi, 1995)	*k* = −15→14
*T*_min_ = 0.940, *T*_max_ = 0.991	*l* = −5→5
3464 measured reflections	

### Refinement

**Table:**

Refinement on *F*^2^	Primary atom site location: structure-invariant direct methods
Least-squares matrix: full	Secondary atom site location: difference Fourier map
*R*[*F*^2^ > 2σ(*F*^2^)] = 0.036	Hydrogen site location: inferred from neighbouring sites
*wR*(*F*^2^) = 0.089	H atoms treated by a mixture of independent and constrained refinement
*S* = 1.00	*w* = 1/[σ^2^(*F*_o_^2^) + (0.0522*P*)^2^] where *P* = (*F*_o_^2^ + 2*F*_c_^2^)/3
659 reflections	(Δ/σ)_max_ < 0.001
94 parameters	Δρ_max_ = 0.22 e Å^−^^3^
1 restraint	Δρ_min_ = −0.19 e Å^−^^3^

### Special details

**Table:**

Geometry. All e.s.d.'s (except the e.s.d. in the dihedral angle between two l.s. planes) are estimated using the full covariance matrix. The cell e.s.d.'s are taken into account individually in the estimation of e.s.d.'s in distances, angles and torsion angles; correlations between e.s.d.'s in cell parameters are only used when they are defined by crystal symmetry. An approximate (isotropic) treatment of cell e.s.d.'s is used for estimating e.s.d.'s involving l.s. planes.
Refinement. Refinement of *F*^2^ against ALL reflections. The weighted *R*-factor *wR* and goodness of fit *S* are based on *F*^2^, conventional *R*-factors *R* are based on *F*, with *F* set to zero for negative *F*^2^. The threshold expression of *F*^2^ > σ(*F*^2^) is used only for calculating *R*-factors(gt) *etc*. and is not relevant to the choice of reflections for refinement. *R*-factors based on *F*^2^ are statistically about twice as large as those based on *F*, and *R*- factors based on ALL data will be even larger.

### Fractional atomic coordinates and isotropic or equivalent isotropic displacement parameters (Å^2^)

**Table:**

	*x*	*y*	*z*	*U*_iso_*/*U*_eq_	
O1	0.32520 (17)	0.52017 (15)	0.4700 (5)	0.0250 (5)	
N1	0.53348 (19)	0.80213 (18)	0.7377 (6)	0.0200 (5)	
N2	0.4393 (2)	0.85281 (17)	0.5557 (6)	0.0219 (5)	
N3	0.3276 (2)	0.80311 (18)	0.5797 (6)	0.0213 (5)	
N4	0.34795 (18)	0.71697 (17)	0.7884 (6)	0.0177 (5)	
N5	0.2526 (2)	0.6400 (2)	0.8533 (6)	0.0189 (5)	
N6	0.5250 (2)	0.64294 (19)	1.0818 (6)	0.0210 (5)	
C1	0.4751 (2)	0.7167 (2)	0.8815 (6)	0.0178 (6)	
C2	0.2470 (2)	0.5459 (2)	0.6773 (7)	0.0203 (6)	
H2	0.1769	0.4961	0.7180	0.024*	
H5N	0.196 (3)	0.661 (2)	0.986 (9)	0.028 (8)*	
H6A	0.607 (3)	0.645 (2)	1.134 (8)	0.043 (10)*	
H6B	0.473 (3)	0.596 (2)	1.192 (8)	0.028 (9)*	

### Atomic displacement parameters (Å^2^)

**Table:**

	*U*^11^	*U*^22^	*U*^33^	*U*^12^	*U*^13^	*U*^23^
O1	0.0209 (11)	0.0284 (10)	0.0259 (11)	−0.0004 (8)	0.0050 (9)	−0.0016 (9)
N1	0.0113 (10)	0.0261 (12)	0.0226 (13)	0.0013 (8)	0.0011 (10)	0.0021 (11)
N2	0.0133 (10)	0.0263 (12)	0.0262 (14)	0.0021 (9)	0.0010 (11)	0.0016 (11)
N3	0.0137 (11)	0.0257 (11)	0.0247 (13)	0.0006 (8)	0.0006 (10)	0.0051 (11)
N4	0.0093 (10)	0.0220 (11)	0.0216 (12)	−0.0009 (8)	0.0009 (10)	0.0012 (10)
N5	0.0090 (10)	0.0269 (12)	0.0209 (13)	−0.0011 (8)	0.0050 (9)	0.0021 (10)
N6	0.0116 (10)	0.0265 (12)	0.0250 (14)	−0.0001 (9)	−0.0023 (10)	0.0025 (11)
C1	0.0107 (11)	0.0229 (13)	0.0198 (15)	0.0027 (9)	0.0004 (11)	−0.0028 (11)
C2	0.0143 (12)	0.0233 (15)	0.0232 (13)	−0.0014 (10)	−0.0013 (11)	0.0058 (13)

### Geometric parameters (Å, °)

**Table:**

O1—C2	1.224 (3)	N5—C2	1.356 (4)
N1—C1	1.335 (3)	N5—H5N	0.84 (4)
N1—N2	1.374 (3)	N6—C1	1.328 (4)
N2—N3	1.294 (3)	N6—H6A	0.87 (4)
N3—N4	1.376 (3)	N6—H6B	0.90 (3)
N4—C1	1.359 (3)	C2—H2	0.9500
N4—N5	1.374 (3)		
			
C1—N1—N2	106.4 (2)	C1—N6—H6A	121 (2)
N3—N2—N1	111.7 (2)	C1—N6—H6B	121 (2)
N2—N3—N4	105.4 (2)	H6A—N6—H6B	117 (3)
C1—N4—N5	128.4 (2)	N6—C1—N1	129.2 (2)
C1—N4—N3	109.3 (2)	N6—C1—N4	123.6 (2)
N5—N4—N3	122.0 (2)	N1—C1—N4	107.2 (2)
C2—N5—N4	119.1 (2)	O1—C2—N5	125.0 (2)
C2—N5—H5N	126.0 (19)	O1—C2—H2	117.5
N4—N5—H5N	114.4 (19)	N5—C2—H2	117.5
			
C1—N1—N2—N3	−0.2 (3)	N2—N1—C1—N4	−0.4 (3)
N1—N2—N3—N4	0.7 (3)	N5—N4—C1—N6	−6.3 (4)
N2—N3—N4—C1	−1.0 (3)	N3—N4—C1—N6	−179.7 (2)
N2—N3—N4—N5	−174.8 (2)	N5—N4—C1—N1	174.2 (3)
C1—N4—N5—C2	−81.1 (3)	N3—N4—C1—N1	0.9 (3)
N3—N4—N5—C2	91.4 (3)	N4—N5—C2—O1	3.8 (4)
N2—N1—C1—N6	−179.9 (3)		

### Hydrogen-bond geometry (Å, °)

**Table:**

*D*—H···*A*	*D*—H	H···*A*	*D*···*A*	*D*—H···*A*
N5—H5N···N1^i^	0.84 (3)	2.02 (3)	2.851 (4)	168 (3)
N6—H6A···O1^ii^	0.87 (3)	2.54 (3)	2.981 (4)	113 (2)
N6—H6A···N3^iii^	0.87 (3)	2.35 (3)	3.164 (4)	156 (3)
N6—H6B···O1^iv^	0.91 (3)	2.12 (3)	3.006 (4)	167 (2)
C2—H2···N2^v^	0.95	2.53	3.404 (5)	152

Symmetry codes: (i) *x*−1/2, −*y*+3/2, *z*+1/2; (ii) −*x*+1, −*y*+1, *z*+1; (iii) *x*+1/2, −*y*+3/2, *z*+1/2; (iv) *x*, *y*, *z*+1; (v) −*x*+1/2, *y*−1/2, *z*+1/2.
